# A novel algorithm for small object detection based on YOLOv4

**DOI:** 10.7717/peerj-cs.1314

**Published:** 2023-03-22

**Authors:** Jiangshu Wei, Gang Liu, Siqi Liu, Zeyan Xiao

**Affiliations:** College of Information Engineering, Sichuan Agricultural University, Ya’an, Sichuan, China

**Keywords:** Deep learning, Convolution neural network, Small object detection, YOLOv4, Feature fusion, Attention mechanisms

## Abstract

Small object detection is one of the difficulties in the development of computer vision, especially in the case of complex image backgrounds, and the accuracy of small object detection still needs to be improved. In this article, we present a small object detection network based on YOLOv4, which solves some obstacles that hinder the performance of traditional methods in small object detection tasks in complex road environments, such as few effective features, the influence of image noise, and occlusion by large objects, and improves the detection of small objects in complex background situations such as drone aerial survey images. The improved network architecture reduces the computation and GPU memory consumption of the network by including the cross-stage partial network (CSPNet) structure into the spatial pyramid pool (SPP) structure in the YOLOv4 network and convolutional layers after concatenation operation. Secondly, the accuracy of the model on the small object detection task is improved by adding a more suitable small object detection head and removing one used for large object detection. Then, a new branch is added to extract feature information at a shallow location in the backbone part, and the feature information extracted from this branch is fused in the neck part to enrich the small object location information extracted by the model; when fusing feature information from different levels in the backbone, the fusion weight of useful information is increased by adding a weighting mechanism to improve detection performance at each scale. Finally, a coordinated attention (CA) module is embedded at a suitable location in the neck part, which enables the model to focus on spatial location relationships and inter-channel relationships and enhances feature representation capability. The proposed model has been tested to detect 10 different target objects in aerial images from drones and five different road traffic signal signs in images taken from vehicles in a complex road environment. The detection speed of the model meets the criteria of real-time detection, the model has better performance in terms of accuracy compared to the existing state-of-the-art detection models, and the model has only 44M parameters. On the drone aerial photography dataset, the average accuracy of YOLOv4 and YOLOv5L is 42.79% and 42.10%, respectively, while our model achieves an average accuracy (mAP) of 52.76%; on the urban road traffic light dataset, the proposed model achieves an average accuracy of 96.98%, which is also better than YOLOv4 (95.32%), YOLOv5L (94.79%) and other advanced models. The current work provides an efficient method for small object detection in complex road environments, which can be extended to scenarios involving small object detection, such as drone cruising and autonomous driving.

## Introduction

In recent years, computer vision techniques based on deep learning have gained significant momentum with the growth of Internet data and the dramatic increase in computer hardware performance. Although the detection accuracy and efficiency of currently used object detection algorithms have improved significantly compared to previous algorithms, the repeated use of convolutional and pooling layers by convolutional neural networks to extract deep semantic information, filtering out pixel points of small objects in the process, has led to poor performance of these algorithms in detecting small objects (Li et al., 2022). For object detection tasks, the society of photo-optical instrumentation engineers (SPIE) defines a small object as a target object with a pixel area of less than 0.12% of the entire image (Zhang, Cong & Wang, 2003). The Microsoft COCO dataset defines a small object as a target object with a pixel area of less than 32 × 32 (Lin et al., 2014). Small objects are difficult to detect due to indistinguishable features, low resolution, complex background, and limited contextual information (Liu et al., 2021). Small object detection is, therefore, more challenging than normal object detection, and so far, good solutions are still rare. Most of the current small object detection methods that perform well improve small object detection performance by optimizing on top of existing object detection algorithms for deep learning. Based on R-CNN, Chen et al. (2016) proposed to enhance the small object detection performance of the model through a contextual model and a small region suggestion generator. Li et al. (2017) proposed the use of generative adversarial networks to generate super-resolution feature representations for small objects in order to reduce the difference in feature representation between small and large objects and thus improve small object detection performance. Hu et al. (2018) proposed a method to extract and fuse feature information at multiple scales, taking advantage of the rich feature information in the deeper networks while considering the small object feature information contained in the shallow networks. Kisantal et al. (2019) proposed to improve the model’s small object detection performance by performing data augmentation on small objects in the dataset. Although the above work has improved detection performance to some extent in small object detection tasks, it also has problems such as great computational effort and vulnerability to complex background interference.

Recently, the you only look once (YOLO) algorithm (Redmon et al., 2016; Redmon & Farhadi, 2017; Redmon & Farhadi, 2018; Bochkovskiy, Wang & Liao, 2020) has been proposed, which performs regression directly to detect objects in images without using RPN, significantly improving the detection speed. Although the representative YOLOv4 model has been applied to a variety of detection tasks, the YOLOv4 model has some inadequacies when targeting challenging detection tasks. Therefore, in solving challenging problems, it is often necessary to improve existing models to achieve better performance of the model in the task. Roy, Bose & Bhaduri (2022) proposed an improved YOLOv4 network framework to solve some traditional challenges in plant disease detection; Roy et al. (2022) proposed a YOLOv4-based WilDect-YOLO network to improve endangered wildlife detection accuracy; Roy & Bhaduri (2022) proposed Dense-YOLOv4 network to detect different growth cycles of mangoes in orchard environment, which provides an effective reference for orchard yield estimation and intelligent drug application.

To address the effects of few effective features, the influence of image noise, and occlusion by large objects on detection accuracy in small object detection tasks, we have improved YOLOv4, a one-stage detection network with detection speed advantages, to optimize the detection speed and accuracy of the model while reducing the number of model parameters. The performance of the improved model is verified by detecting small objects in various challenging environments. The contributions of this article are as follows: First, we include the CSP structure (Wang et al., 2020) into the SPP (He et al., 2015), CBL × n in the PANet (Liu et al., 2018) module of the YOLOv4 network to make the network lightweight while maintaining sufficient detection accuracy and use the SiLU activation function with better smoothness to enhance the model the non-linear feature learning capability. Second, we add a new branch after CSP2 to extract the shallow network feature information and increase the depth of the neck part to fuse the feature information extracted from the added branch. Then, the 19 × 19 detection head is removed from the three detection heads, and a new 160 × 160 detection head is added. The modified detection head is used to predict the fused feature information, which improves the network’s ability to extract and detect small object location information. Third, when concatenating feature information, the trainable fusion weights are increased to optimize the fusion effect of different levels of feature information. Fourth, the coordinate attention (CA) mechanism (Hou, Zhou & Feng, 2021) is used in CSP_CBS (modified CBL × n) to focus on the details of the features containing location relationships to further optimize the feature fusion results. In addition, the improved network has fewer parameters, better performance for small object detection than the state-of-the-art detection model, and a detection speed that meets the criteria for real-time object detection.

This article is organized as follows: “Materials and Methods” introduces the dataset, the YOLOv4 network, and our proposed network; “Experimental results and discussion” describes our experimental setup, performance metrics, experimental procedure, and related discussions; “Conclusions” summarizes the conclusions and outlook of the current work.

## Materials and Methods

### Data source

The experiments in this article use the VisDrone2019 dataset (Du et al., 2019) and the S2TLD dataset (Yang et al., 2022). The VisDrone2019 dataset was collected by the AISKYEYE team at the Lab of Machine Learning and Data Mining, Tianjin University, China. The dataset contains a large number of objects in urban and rural road scenes (10 categories such as pedestrians, vehicles, bicycles, *etc*.), covering a wide variety of scenes and containing a large number of small objects. The dataset was collected by the drone in different scenarios, weather, and lighting conditions, therefore, the background of the object to be detected is more complex. The S2TLD dataset contains five categories (red, yellow, green, off, and waiting). It covers a wide range of road scenarios, such as busy street scenes, multiple visible traffic lights, and traffic tail lights that may be confused with traffic lights (*e.g*., large circular tail lights, *etc*.). This experiment randomly partitioned the 7,019 labeled images from the VisDrone2019 dataset in a ratio close to 7:2:1, yielding a training set of 4,908 images, a validation set of 1,409 images, and a test set of 702 images. The 5,786 labeled images of the small traffic light dataset were divided in the same way, yielding 4,045 images for the training set, 1,162 images for the validation set, and 579 images for the test set.

### YOLOv4 network

**Figure 1:**
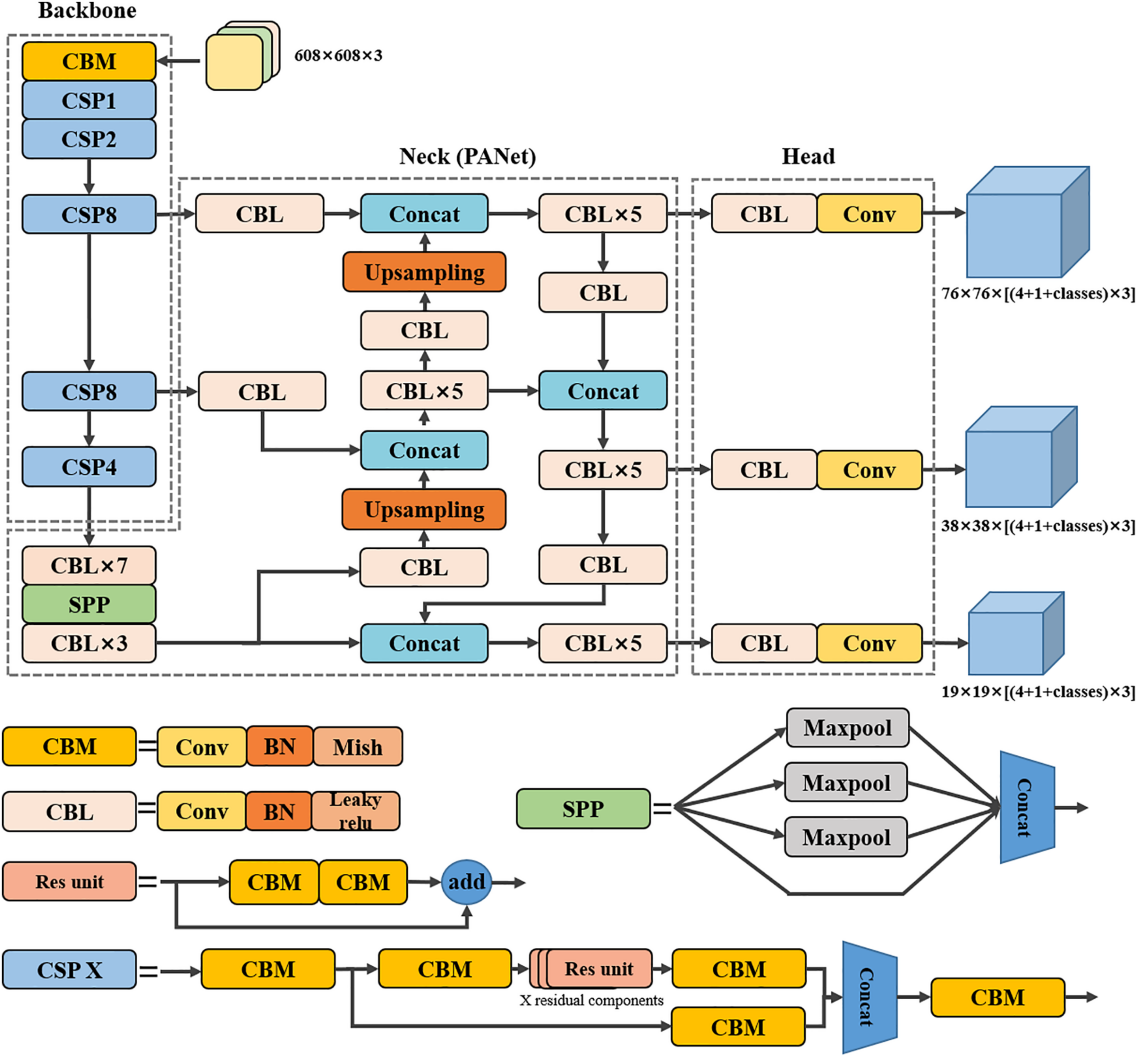
Network structure diagram of YOLOv4.

The YOLOv4 network is an object detection algorithm proposed by Bochkovskiy, Wang & Liao (2020) based on YOLOv3, whose network structure mainly consists of three parts: backbone, neck, and detection head. The YOLOv4 structure diagram is shown in Fig. 1. As can be seen in Fig. 1, the YOLOv4 network is mainly composed of a combination of convolution, batch normalization (Ioffe & Szegedy, 2015), and activation functions (Mish (Misra, 2019) and Leaky ReLU (Maas, Hannun & Ng, 2013)). The backbone part is the CSPDarknet53 network, which is a structure based on yolov3’s Darknet53 network structure with the introduction of the CSP module to ensure the accuracy of the backbone network in extracting feature information while reducing the amount of computation and is composed of CBM, CSP1, CSP2, CSP8, CSP8, and CSP4 in Fig. 1. The neck part uses a spatial pyramidal pooling (SPP) structure to extract backbone network feature information and increase the model receptive field. Moreover, based on the feature pyramid networks (FPN) (Lin et al., 2017a) of the YOLOv3 network, YOLOv4 uses the PANet structure for feature fusion, so that shallow and deep feature information can be better fused. Finally, compared to the Leaky ReLU activation function used in the backbone of the YOLOv3 network, the YOLOv4 network uses a smoother Mish activation function in the backbone, which allows better information to penetrate deeper into the neural network, resulting in better accuracy and generalization. In the detection head part, YOLOv4 continues to use the three detection heads of YOLOv3. The input 608 × 608 × 3 scale image to be detected is firstly extracted feature information by the backbone part, followed by the semantic representation of features by the neck part. Finally, the predicted feature maps of 19 × 19, 38 × 38, and 76 × 76 scales are obtained by the CBL module and convolution operation for the prediction of large, medium, and small objects. In addition, YOLOv4 optimizes the calculation method of predicted object center coordinates in YOLOv3 by introducing scaling factors, which solves the problem that the predicted coordinates are difficult to predict the real coordinates when the real object centroid is very close to the grid boundary in the predicted feature map.

### The proposed model network structure

Although the above techniques can effectively improve the detection accuracy of the model, the original YOLOv4 can only provide limited detection accuracy in small object detection tasks due to the low resolution of the object to be detected, the small amount of available information, and the complex background. In addition, YOLOv4 has a large number of parameters and is not suitable for mobile devices.

**Figure 2:**
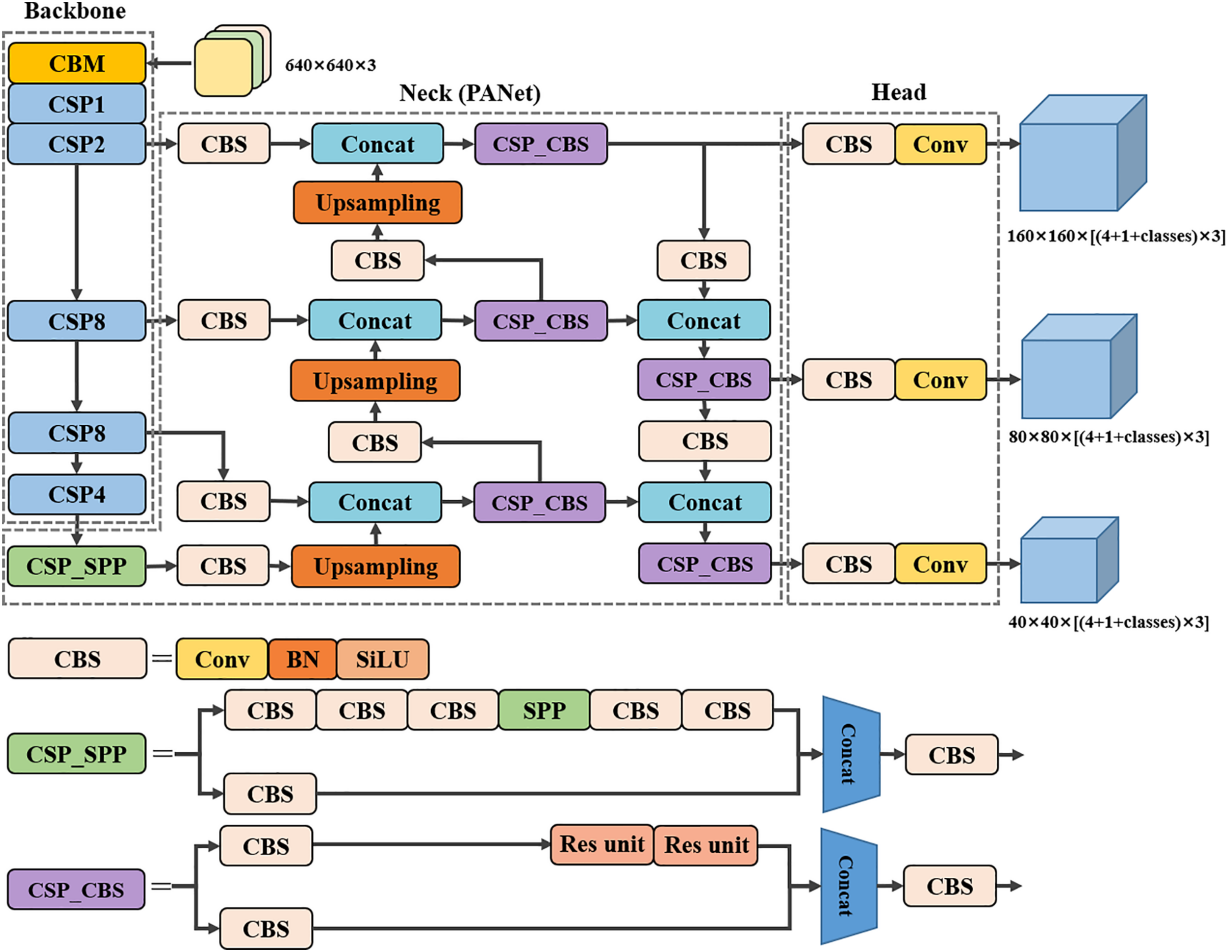
Network structure diagram of the proposed model.

In order to solve the problems associated with the above small object detection task, the YOLOv4 algorithm is improved and optimized in this article to achieve accurate prediction of small object detection in complex background environments. The structure of the improved network architecture is shown in Fig. 2, and each modification is discussed in this section. The proposed modifications are divided into four aspects: using the CSP structure in the SPP and CBL of the original neck part and using the SiLU activation function with better smoothness in the whole neck part; adding a convolutional layer to extract additional feature information after the CSP2 of the original backbone part and expanding the depth of the neck part to fuse the additional extracted feature information, adding a small object detection head and removing the 19 × 19 scale detection head; adding learnable weights to Concat in the neck part to optimize the feature fusion results; introducing an attention mechanism in the CBS of the neck part to improve the model’s ability to focus on the location information. It is found that with the above modifications, the model outperforms other advanced detection models in the small object detection task.

#### The neck part uses a CSP structure

**Figure 3:**
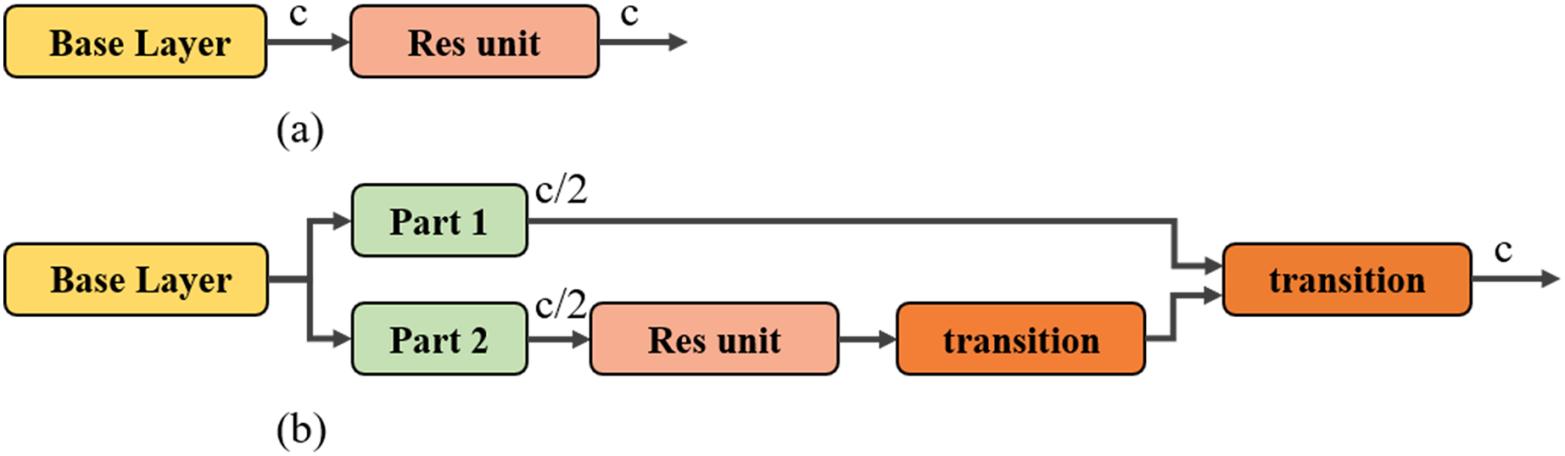
Structure diagram of CSP: (A) residual module, (B) residual module with CSP structure, transition module: convolution block to adjust the height, width, and number of channels of the feature map.

In this subsection we introduce SPP and CBL from the original YOLOv4 neck into the CSP structure. We use the CSP architecture in the neck module of the original YOLOv4 to lighten the network and improve the network detection speed by exploiting the ability of the CSP architecture to enrich the gradient combination and reduce the computational effort. This improved model will be referred to as Version-1 in the following section. The residual module (He et al., 2016) without the CSP structure is shown in Fig. 3A, where the number of input and output channels is c. The residual module with the CSP structure is shown in Fig. 3B, where the input channel c of the residual module is changed to c/2 by the Part1 and Part2 modules. Then the output of the whole module is adjusted to c by the subsequent transition operation (convolution block to adjust the height, width, and number of channels of the feature map). Since the number of input channels of the Part2 module is c/2, the number of parameters in the residual module part is reduced. The presence of the Part1 module, on the other hand, enables the structure to have a richer combination of gradients.

**Figure 4:**
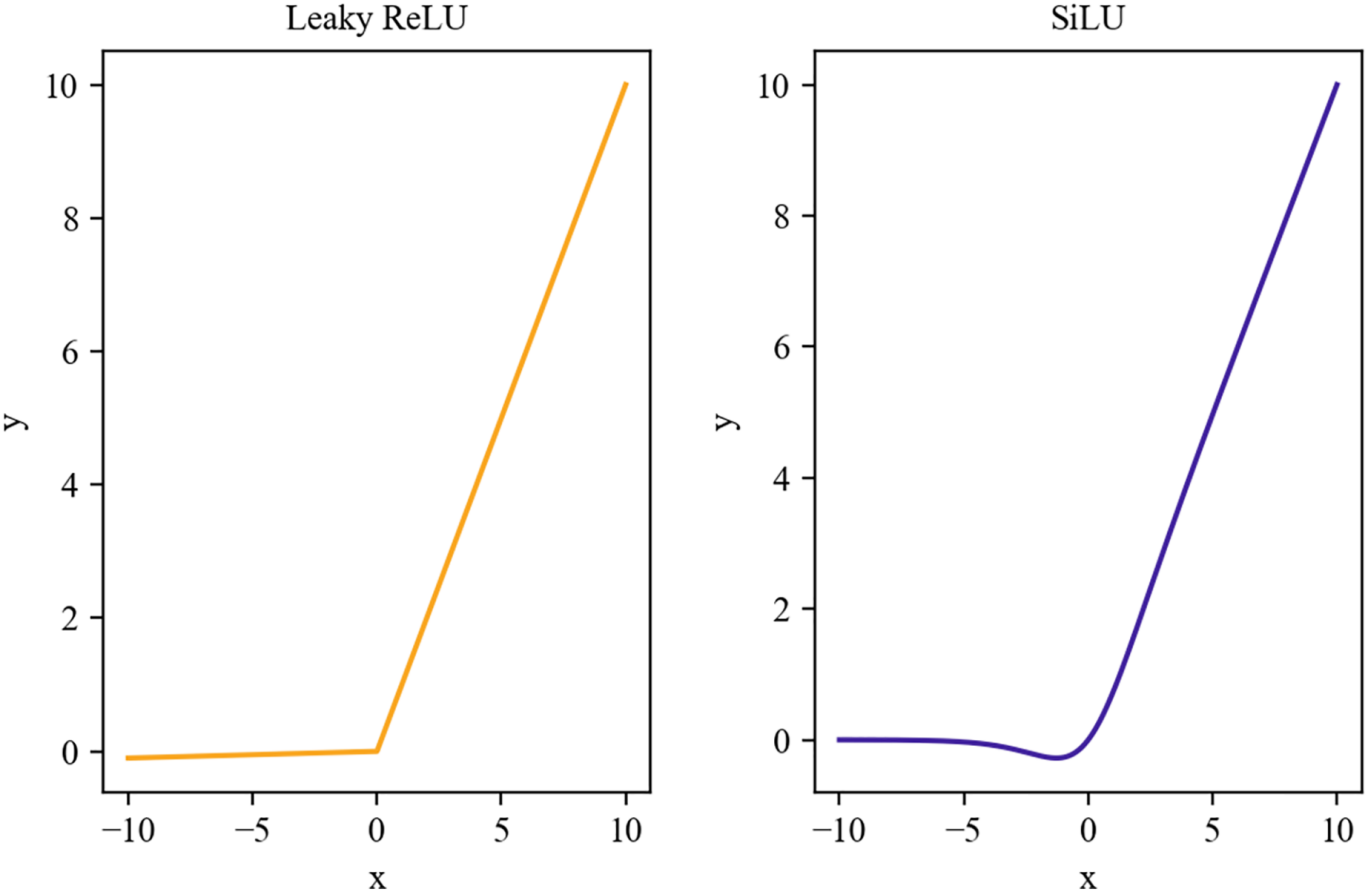
Leaky ReLU and SiLU activation functions.

As can be seen in Fig. 4, both the Leaky Relu activation function and the SiLU activation function (Ramachandran, Zoph & Le, 2017) retain some values of the negative axis, which allows the negative axis information not to be lost in its entirety, so that the phenomenon of dead neurons does not occur when using them as in the case of the ReLU activation function.

The activation function used in the neck part of YOLOv4 is the Leaky ReLU activation function, with the formula shown in Eq. (1), which applies different functions in the positive and negative intervals, which results in its inability to provide consistent relational predictions for positive and negative input values. Therefore, we use the SiLU activation function with better smoothness as the neck part activation function to enhance the model’s generalization ability, and the SiLU activation function is shown in Eq. (2).

(1) $$Leaky\,ReLU=\left\{ {\matrix{ {x,} & {x > 0} \cr {0.01x,} & {x \le 0} \cr } }, \right.$$

(2) $$SiLU=\displaystyle{x \over {1 + {e^{ - x}}}}.$$

#### Improved detection head for YOLOv4 network

After the modifications to YOLOv4 in the previous subsection, we achieved some performance improvements, which will be discussed later in the experiments. Therefore, the following three proposed modifications will be based on the Version-1 model. The original YOLOv4 network supports input images with a maximum size of 608 × 608. When the 608 × 608 size image is input to the network to reach the detection head position, the output feature map has three scales of height and width of 19 × 19, 38 × 38, and 76 × 76, respectively, which are used to detect large, medium, and small objects in the image. Firstly, in order to better extract the small object information from the image and, at the same time, ensure the detection speed of the network, this article changes the input image size of the network to 640 × 640, which not only increases the image resolution information obtained by the network, but also has little impact on the detection speed of the network because the input image size is only 640 × 640 after the increase. Although the 20 × 20 scale prediction feature map output by the detection head contains richer semantic information, the prediction feature map at this scale is less capable of extracting small object location information due to its high number of downsampling. To improve the model’s ability to extract small object position information, we removed the 20 × 20 scale detection head and added a 160 × 160 scale detection head. Our approach is to add a feature fusion path after the shallow module CSP2 in the backbone part of the original model, which is capable of extracting more location information, and then expand the number of layers of PANet and finally combine the new path, the expanded feature fusion module and the new 160 × 160 scale detection head to form a network with more capability of extracting small object location information, as shown in Fig. 2. Specifically, we added a CBS module to extract the feature information extracted by CSP2, and increased the number of feature fusion layers by adding a new CBS module, upsampling, and downsampling, then modifying the detection header. We refer to this modified model as Version-2.

#### Improved feature fusion module

**Figure 5:**
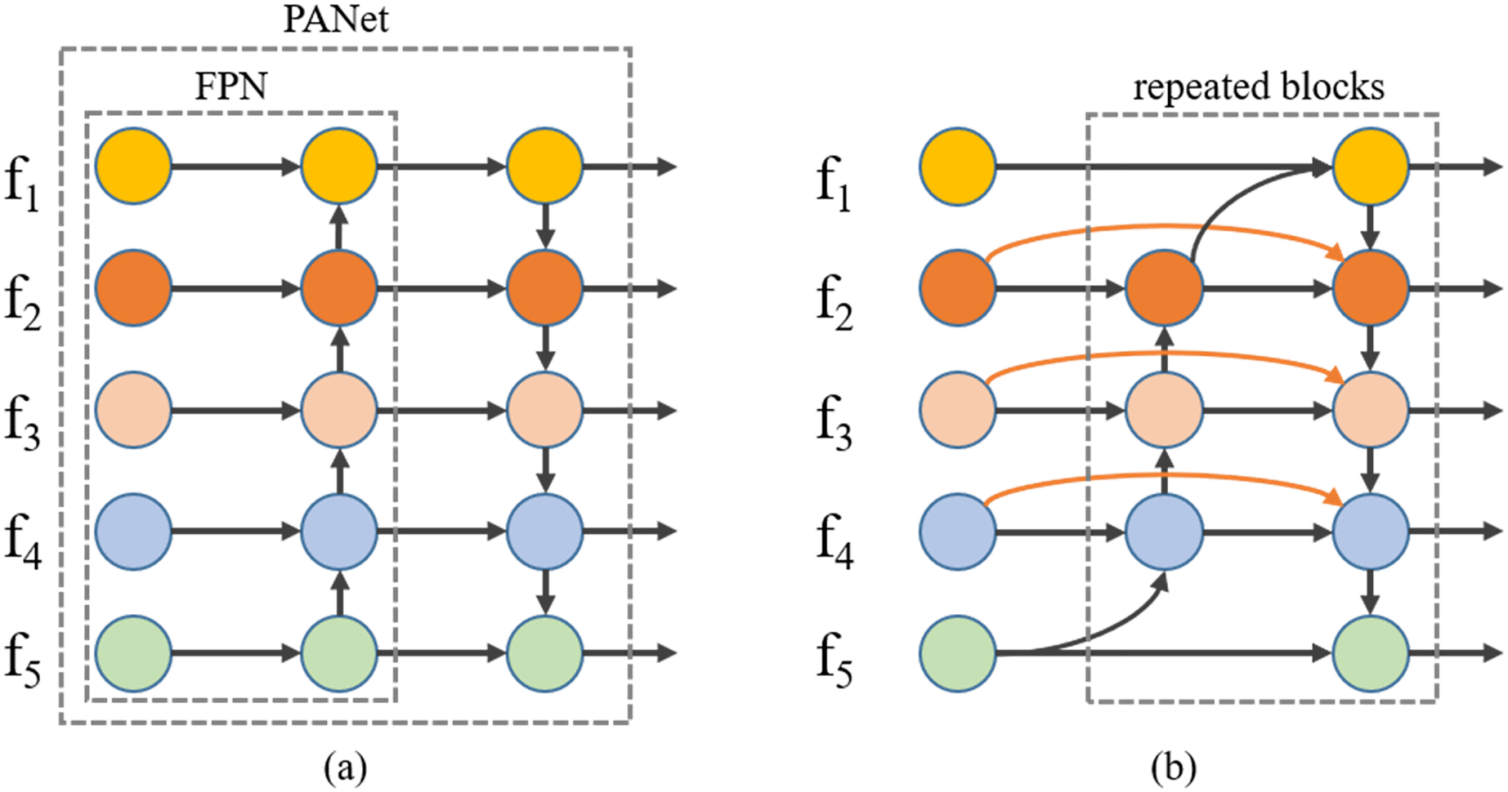
Different feature fusion structures: (A) FPN and PANet structure, (B) BiFPN structure.

Compared with the traditional feature pyramid network (FPN) structure, which is restricted by unidirectional information flow, PANet adds a path to the FPN structure for a secondary fusion of model feature information, thus making better use of the location information of shallow features, improving model performance and demonstrating the effectiveness of bidirectional fusion. The FPN structure and PANet structure are shown in Fig. 5A, where ${{\rm f}_{\rm 1}}$–${{\rm f}_{\rm 5}}$ represent multi-scale features from shallow to deep. To give a specific example, if ${{\rm f}_{\rm 1}}$ has an input resolution of 320 × 320, ${{\rm f}_{\rm 2}}$ has an input resolution of 160 × 160, and so on, and ${{\rm f}_{\rm 5}}$ has an input resolution of 20 × 20, ${{\rm f}_{\rm 1}}$–${{\rm f}_{\rm 5}}$, after feature fusion by FPN, outputs a resolution rule as shown in Eqs. (3)–(7).

(3) $${\rm f}_5^{out} = Conv ({\rm f}_5^{in}),$$

(4) $${\rm f}_4^{out} = Conv ({\rm f}_4^{in} + Resize ({\rm f}_5^{out})),$$

(5) $${\rm f}_3^{out} = Conv ({\rm f}_3^{in} + Resize ({\rm f}_4^{out})),$$

(6) $${\rm f}_2^{out} = Conv ({\rm f}_2^{in} + Resize ({\rm f}_3^{out})),$$

(7) $${\rm f}_1^{out} = Conv ({\rm f}_1^{in} + Resize ({\rm f}_2^{out})).$$

In Eqs. (3)–(7), Resize is usually an upsampling or downsampling operation for resolution matching, Conv is usually a convolutional operation for feature processing, and ${\rm f}_{\rm 4}^{{\rm out}}$ is the output feature at level 4 on the bottom-up pathway. All other features are constructed similarly.

We studied PANet in depth and found that its bi-directional fusion mechanism is relatively simple, with only one bottom-to-top path and one top-to-bottom path. To improve the performance of the feature fusion part, we used a weighted bidirectional feature pyramid network (BiFPN) (Tan, Pang & Le, 2020) to replace PANet as the new feature fusion structure. BiFPN uses each bi-directional (bottom-up and top-down) path as a feature network layer, repeated multiple times, and uses learnable fusion weights with a richer fusion hierarchy and more efficient fusion mechanisms, as shown in Fig. 5B. To give a specific example of the same, if ${{\rm f}_{\rm 1}}$ has an input resolution of 320 × 320, ${{\rm f}_{\rm 2}}$ has an input resolution of 160 × 160, and so on, and ${{\rm f}_{\rm 5}}$ has an input resolution of 20 × 20, ${{\rm f}_{\rm 1}}$–${{\rm f}_{\rm 5}}$, after feature fusion by BiFPN, outputs a resolution rule as shown in Eqs. (8) and (9).

(8) $${\rm f}_4^{mid} = Conv \left(\displaystyle{{{w_{41}} \cdot {\rm f}_4^{in} + {w_{42}} \cdot Resize ({\rm f}_5^{in})} \over {{w_{41}} + {w_{42}} + \varepsilon }}\right),$$

(9) $${\rm f}_4^{out} = Conv \left(\displaystyle{{{{{w}^{\prime}}_{41}} \cdot {\rm f}_4^{in} + {{{w}^{\prime}}_{42}} \cdot {\rm f}_4^{mid} + {{{w}^{\prime}}_{43}} \cdot Resize ({\rm f}_3^{out})} \over {{{{w}^{\prime}}_{41}} + {{{w}^{\prime}}_{42}} + {{{w}^{\prime}}_{43}} + \varepsilon }}\right).$$

In Eqs. (8) and (9), ${\rm f}_{\rm 4}^{{\rm mid}}$ is the intermediate feature at level 4 on the bottom-up pathway, and ${\rm f}_{\rm 4}^{{\rm out}}$ is the output feature at level 4 on the top-down pathway. $\omega$ is the weighting parameter introduced, and ε is taken as a fixed value of 0.0001 to avoid numerical instability. All other features are constructed in a similar manner.

To avoid overly complex feature fusion structures affecting the detection speed of the network, we decided to use the bi-directional fusion structure of the BiFPN only once and keep the number of intermediate feature layers in the BiFPN the same as the number of intermediate feature layers in the PANet. In addition, we find that the fusion using Add operation is not satisfactory, so we add new trainable fusion weights in Concat to improve the effect of feature fusion. Again, we refer to this modified model as Version-3.

#### Embedded coordinate attention (CA) module in the feature fusion structure

**Figure 6:**
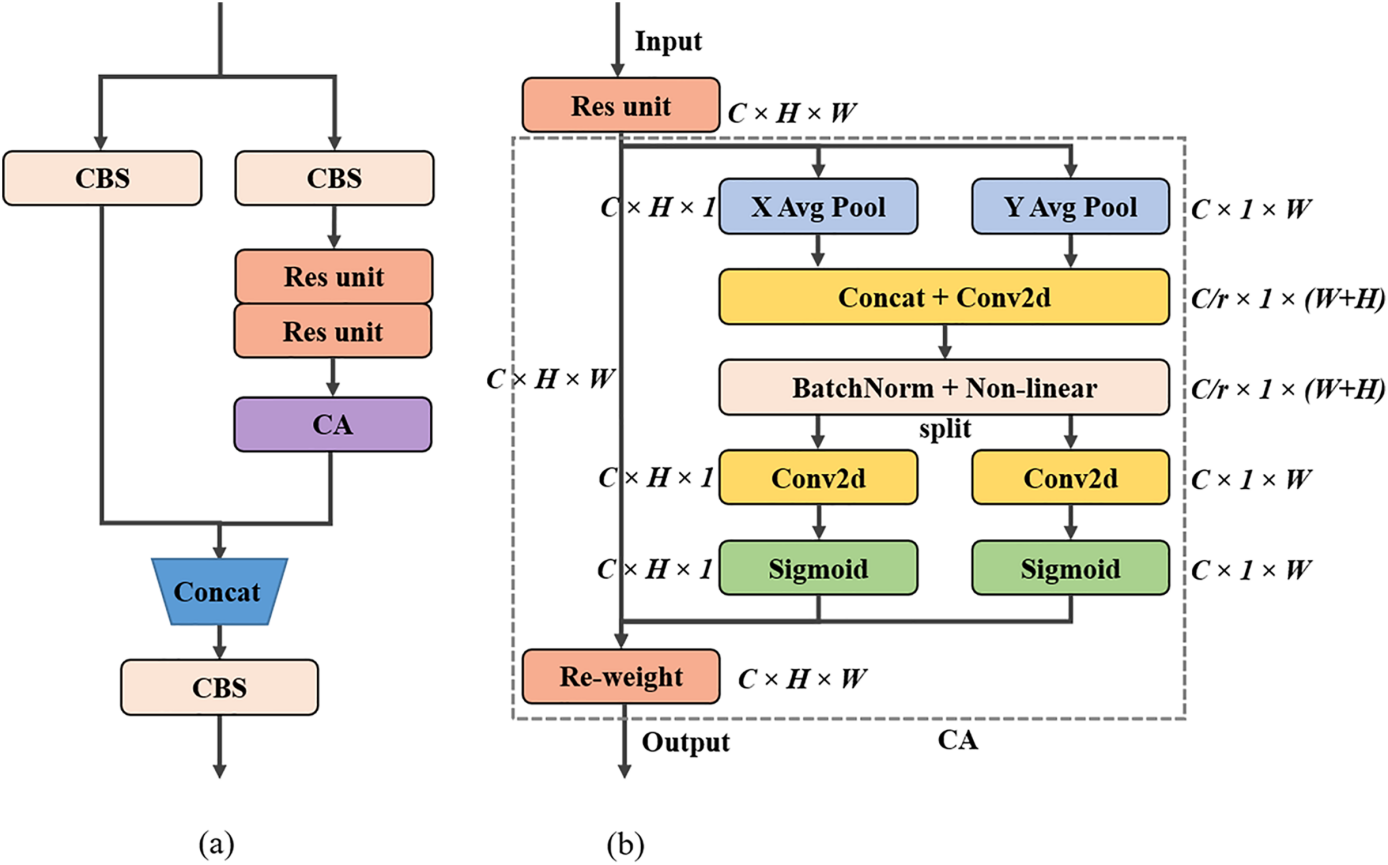
Attention mechanism related modules: (A) residual module structure diagram embedded in CA module, (B) CA module structure diagram.

After the previous modifications, we consider using the attention mechanism to improve the model performance. In the field of deep learning, models often need to receive and process large amounts of data. However, only a small portion of certain data is usually important at a given moment. This situation is ideal for using the attention mechanism to make the model focus on what we want. Therefore, this article introduces an attention mechanism in the neck part of the model so that the model can pay extra attention to the location information of small objects when performing feature fusion, thus improving the performance of the whole model. Among the currently used attention mechanisms, squeeze-and-excitation (SE) module (Hu, Shen & Sun, 2018) focuses on extracting inter-channel information and ignores location information. While convolutional block attention module (CBAM) (Woo et al., 2018) extracts location attention information, it cannot extract long-distance relationships. In the end, we decided to select the CA module to help improve the performance of our model. The residual structure of the embedded CA module designed in this article is shown in Fig. 6A, and the CA module encodes channel relations and long-range dependencies with precise position information in two steps: coordinate information embedding and coordinate attention generation, as shown in Fig. 6B.

The first step is to embed the coordinate information. In the SE module, the global information embedding is done by the squeeze operation, as shown in Eq. (10).

(10) $${z_c} = \displaystyle{1 \over {H \times W}}\sum\limits_{i = 1}^H {\sum\limits_{j = 1}^W {{x_c}(i,j)} }.$$

In Eq. (10), *H* and *W* denote the height and width of the input feature map. ${{\rm x}_{\rm c}}$ is the c-th channel of the feature map, and ${{\rm z}_{\rm c}}$ is the result of squeezing the global information of the c-th channel of that feature map. SE modules using this type of compression have difficulty focusing on the location information in the features. The CA module decomposes this compression method into a pair of one-dimensional feature encoding operations, *i.e*., each channel is encoded along horizontal and vertical coordinates by two spatially scoped pooling kernels (*H*, 1), (1, *W*), respectively, formulated as in Eqs. (11) and (12).

(11) $$z_c^h(h) = \displaystyle{1 \over W}\sum\limits_{0 \le i \le W} {{x_c}(h,i)},$$

(12) $$z_c^w(w) = \displaystyle{1 \over H}\sum\limits_{0 \le j \le H} {{x_c}(j,w)}.$$

Among them, *H* is the height position in the input feature map, and Eq. (11) is the encoded output of the c-th channel of the feature map at height *h*. Similarly, *W* is the width position in the input feature map, and Eq. (12) is the encoded output of the c-th channel of the feature map at width *w*. The compression method used by the CA module aggregates features along two spatial directions, height and width, respectively, to generate a pair of direction-sensitive feature maps that not only capture long-range dependencies in one spatial direction but also retain precise location information in the other, which facilitates the network to locate the object of interest more accurately.

The second step coordinates attention generation. After capturing the location information in the first step, the resulting feature map is stitched together, followed by a 1 × 1 convolution operation to generate an intermediate feature map, as in Eq. (13).

(13) $${\bf f} = \delta ({F_1}([{{\bf z}^h},{{\bf z}^w}])).$$

In Eq. (13), [.,.] is the concatenation operation, ${{\rm F}_{\rm 1}}$ is the 1 × 1 convolution operation, δ is the non-linear activation function, and **f** is the resulting intermediate feature map whose number of channels is 1/r of the number of channels of the input feature map, with the default value of r being 32, which is used to control the scaling. The intermediate feature map **f** is then divided into two independent tensors, ${{\rm f}^{\rm h}}$ and ${{\rm f}^{\rm w}}$, along the spatial dimension, and the two are each passed through a 1 × 1 convolution operation to obtain attention weights ${{\rm g}^{\rm h}}$ and ${{\rm g}^{\rm w}}$ with the same number of channels as the input feature map X, as in Eqs. (14) and (15).

(14) $${{\bf g}^h} = \sigma ({F_h}({{\bf f}^h})),$$

(15) $${{\bf g}^w} = \sigma ({F_w}({{\bf f}^w})).$$

In Eqs. (14) and (15), ${{\rm F}_{\rm h}}$ and ${{\rm F}_{\rm w}}$ are both 1 × 1 convolution operations, and σ is a sigmoid activation function. Finally, ${{\rm g}^{\rm h}}$ and ${{\rm g}^{\rm w}}$ are expanded and weighted with the input feature map X to obtain the output Y of the CA module, as in Eq. (16).

(16) $${y_c}(i,j) = {x_c}(i,j) \times g_c^h(i) \times g_c^w(j).$$

In Eq. (16), ${{\rm x}_{\rm c}}\left( {{\rm i,\; j}} \right)\;$is the information at the input feature map height *h*, width *w*, and channel *c* position, ${\rm g}_{\rm c}^{\rm h}\left( {\rm i} \right)$ is the attention weight corresponding to the input feature map height *h* and channel *c* position, and ${\rm g}_{\rm c}^{\rm w}\left( {\rm j} \right)$ is the attention weight corresponding to the input feature map width *w* and channel *c* position. ${{\rm y}_{\rm c}}\left( {{\rm i,\; j}} \right)$ is the output of the input feature map on channel *c* after passing through the CA module, and Y is the final output of all channels after concatenating them together.

If CA is used globally throughout the model, the number of parameters and detection speed of the modified network will be greatly affected. At the same time, considering the characteristics of the neck part incorporating different levels of feature information, we decide to include the CA module in the CSP_CBS module to enhance the ability of the neck network to focus on the location relationship. We refer to this modified model as Version-4.

## Experimental results and discussion

### Experimental setting

The software environment used for model training in this article is Ubuntu 18.04.3, Conda 4.11.0, Python 3.9.7, and PyTorch 1.10.0. The hardware environment includes an Intel(R) Xeon(R) Gold 6240, 2.6 GHz × 32 processing frequency, and an NVIDIA Tesla V100 graphics card with 32G video memory.

The YOLOv4 network is based on anchors of different sizes and aspect ratios to predict the object. Having the right anchor size and aspect ratio can help the model converge faster and fit the dataset better during training. Therefore, we use the k-means algorithm to re-cluster the anchors of the above two datasets to obtain anchors of suitable size and use the genetic algorithm to vary the length and width of the clustered anchors so that there is a slight variation in the anchors obtained by clustering to increase the diversity and generalization of the anchors. In the data preprocessing stage, we used online data augmentation methods (*e.g*., scaling, panning, color saturation adjustment, *etc*.) (Cubuk et al., 2019; Zhang et al., 2017; DeVries & Taylor, 2017; Yun et al., 2019) to improve the robustness of the model. We also used the letterbox adaptive image scaling technique to adjust the image to a 640 × 640 resolution before the image was fed into the network, thus maintaining the original image aspect ratio and avoiding the loss of image information due to simple and violent resize operations. During the training phase, we adopt a warm-up strategy. That is, we train at a small learning rate at the beginning of the training period to slow down the overfitting of the model to the mini-batch in the initial phase, thus maintaining the stability of the model. The model parameters were updated using the SGD optimizer (Ruder, 2016), setting the initial learning rate to 0.003 when warm-up, 0.01 after the warm-up was completed, and gradually decreasing to 0.0001 after 300 iterations, with a batch size of eight.

To validate the model performance, we calculate the precision (*P*) and recall (*R*) (Powers, 2020) for the test set and use mean average precision (mAP) (Everingham et al., 2010) the number of model parameters, and the time taken to detect a single image as evaluation metrics. The calculation is shown in Eqs. (17)–(19).

(17) $$P{\rm = }\displaystyle{{{T_{\rm P}}} \over {{T_{\rm P}} + {F_{\rm P}}}},$$

(18) $$R = \displaystyle{{{T_{\rm P}}} \over {{T_{\rm P}} + {F_{\rm N}}}},$$

(19) $$mAP = \displaystyle{{\sum\limits_{c = 1}^C {AP(c)} } \over C} \times 100 \rm%.$$

In the above formula Eqs. (17)–(19), ${{\rm T}_{\rm P}}$ is the correctly classified positive sample, ${{\rm F}_{\rm P}}$ is the incorrectly classified negative sample, ${{\rm F}_{\rm N}}$ is the incorrectly classified positive sample, AP is the area under the *P*-*R* curve, and C is the number of categories in the sample.

**Figure 7:**
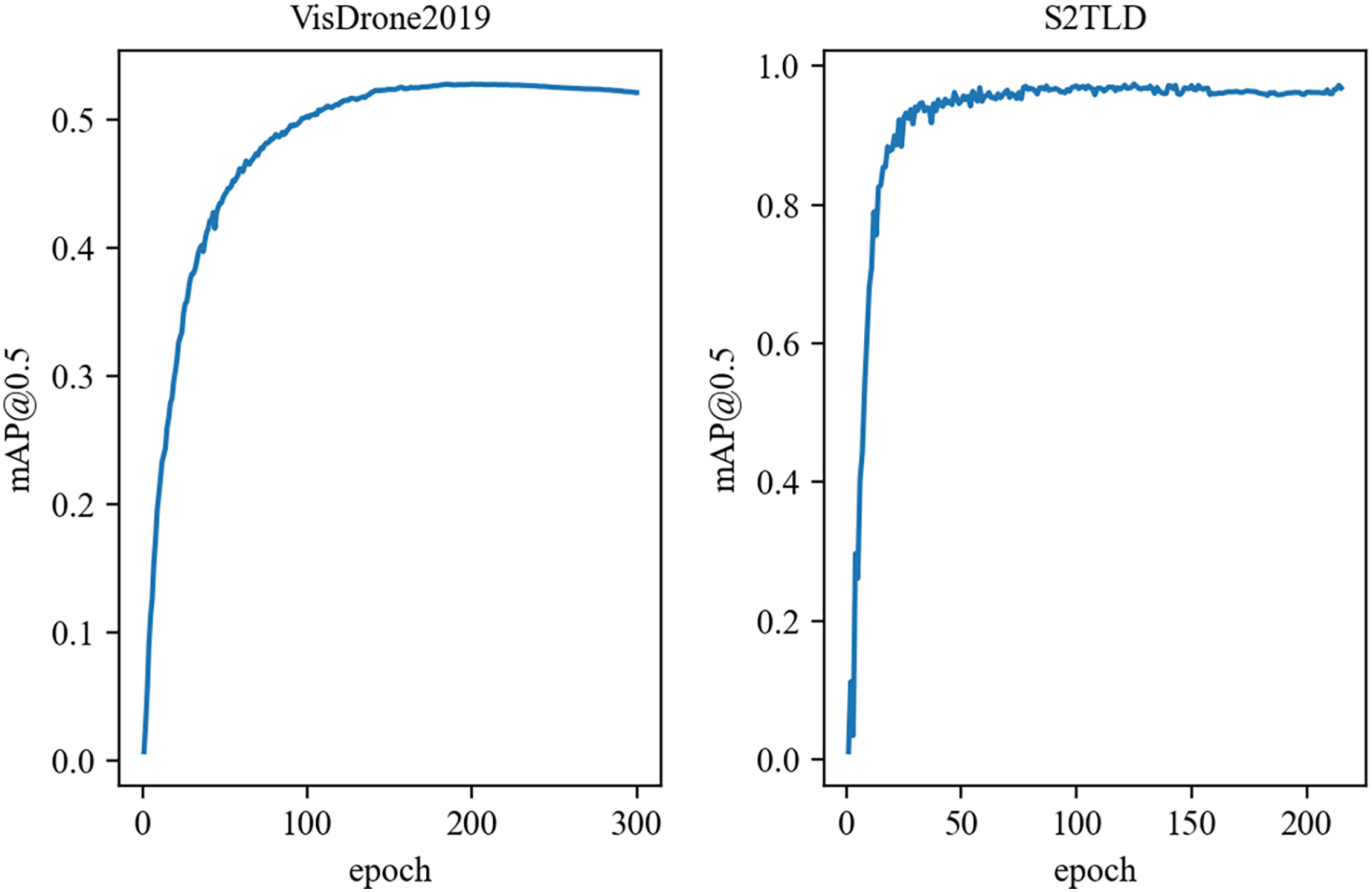
The mAP training curve of the YOLOv4-ours model.

As shown in Fig. 7, the improved YOLOv4 model in this article had converged after 300 iterations on the VisDrone2019 dataset; it converged even faster on the S2TLD dataset, so training was stopped after 215 iterations.

### Lightweight the neck part

To validate the effectiveness of the improved method in this article, we conducted experiments on all scale objects of the VisDrone2019 dataset and S2TLD dataset. First, we optimize the neck part of the original YOLOv4 network using the CSP structure and changing the activation function (Version-1). As can be seen in Table 1, compared to the performance of the original YOLOv4 model on the VisDrone2019 dataset, the Version-1 model has a reduced number of parameters, only 82.1% of the original model, a 0.94% improvement in mAP, and a reduction in inference time from 0.025 to 0.023 s for a single image. Similarly, on the S2TLD dataset, compared to the performance of the original YOLOv4 model, the Version-1 model has a reduced number of parameters, only 82.1% of the original model, some increase in mAP, and faster inference for a single image, as shown in Table 2. These performance gains are not only due to the CSP structure that integrates the gradient changes into the feature map from start to finish, thus ensuring model accuracy while reducing computational effort, but also to the smoother SiLU activation function, which improves the generalization capability of the model.

**Table 1:** Performance comparison of VisDrone2019 dataset after the first improvement.

Model	mAP/%	Parameter/million	t/s
YOLOv4	42.79	63.99	0.025
Version-1	43.73	52.54	0.023

**Table 2:** Performance comparison of S2TLD dataset after the first improvement.

Model	mAP/%	Parameter/million	t/s
YOLOv4	95.32	63.96	0.025
Version-1	95.45	52.51	0.023

### Performance of different feature fusion modules

To validate the effectiveness of the neck part using the new detection head, the BiFPN structure, and the CA module (*i.e*., the improved effect of Version-2, Version-3, and Version-4), in this subsection, we perform ablation experiments using these three feature fusion methods based on the Version-1 model described above. As can be seen in Table 3, on the VisDrone2019 dataset, the model with the new detection head (Version-2) has a high mAP of 52.13% compared to the Version-1 model, and the model also has fewer parameters, with an inference speed of 0.024 seconds for a single image, which is only 0.001 seconds different from the Version-1 model, but also better than the original YOLOv4 model. In another aspect, as shown in Table 4, on the S2TLD dataset, the Version-2 model improves the mAP by 0.98% compared to the Version-1 model, with fewer parameters and faster inference speed. The analysis of the experimental results in Tables 3 and 4 shows that the model using our improved BiFPN structure (Version-3) on the VisDrone2019 dataset and the S2TLD dataset increases the number of parameters by a small amount relative to the Version-1 model and achieves a better mAP while ensuring inference speed. Likewise, the model with the CA module embedded (Version-4) performs well on both datasets, adding a small number of parameters and obtaining a better mAP compared to the Version-1 model.

**Table 3:** Performance comparison of different feature fusion modules in the VisDrone2019 dataset.

Model	New detection head	BiFPN	CA	mAP/%	Parameter/million	t/s
YOLOv4				42.79	63.99	0.025
Version-1				43.73	52.54	**0.023**
Version-2	√			52.13	**44.62**	0.024
Version-3		√		44.04	52.61	**0.023**
Version-4			√	43.93	52.58	0.026
Version-5	√	√		52.40	44.76	0.025
Version-6	√		√	52.51	44.65	0.028
Version-7		√	√	44.06	52.65	0.026
YOLOv4-ours	√	√	√	**52.76**	44.79	0.028

**Note:**

The best results are in bold.

**Table 4:** Performance comparison of different feature fusion modules in the S2TLD dataset.

Model	New detection head	BiFPN	CA	mAP/%	Parameter/million	t/s
YOLOv4				95.32	63.96	0.025
Version-1				95.45	52.51	**0.023**
Version-2	√			96.43	**44.61**	0.024
Version-3		√		95.61	52.58	**0.023**
Version-4			√	95.60	52.56	0.026
Version-5	√	√		96.68	44.74	0.025
Version-6	√		√	96.50	44.64	0.028
Version-7		√	√	96.30	52.62	0.026
YOLOv4-ours	√	√	√	**96.98**	44.77	0.028

**Note:**

The best results are in bold.

Finally, we combine these three different feature fusion methods on the Version-1 model. We find that the combined model has a higher mAP than using them alone on the Version-1 model, suggesting that these three different feature fusion modules bring complementary enhancements to the model and that combining them can further improve model performance. We apply all three feature fusion methods to the Vserion-1 model to form the final model (YOLOv4-ous), which has the highest mAP of 52.76% and 96.98% on the VisDrone2019 and S2TLD datasets, respectively.

### Detection of different small object classes

In this section, we test the original YOLOv4 model and the proposed (YOLOv4-ours) model to further compare the detection performance of the model on different categories to be detected. As shown in Table 5, on the VisDrone2019 dataset, the original YOLOv4 model has a low recall and precision over these 10 target categories, especially on bicycles and awning-tricycles, where there are more serious missed detections and lower detection accuracy. The proposed model in this article improves recall and accuracy over all categories, significantly improving the detection performance on confusingly small objects such as pedestrians and people, bicycles and motorcycles, and tricycles and awning-tricycles.

**Table 5:** Compare the detection performance, detection speed, number of parameters of YOLOv4 and the proposed model (YOLOv4-ours) on the VisDrone2019 dataset.

Model	Class	P/%	R/%	mAP/%	Parameter/million	t/s
YOLOv4	All	53.91	42.88	42.79	63.99	0.025
	Pedestrian	61.66	47.57	50.48		
	People	48.67	31.04	30.76		
	Bicycle	44.01	19.16	19.57		
	Car	70.30	75.44	76.42		
	Van	57.58	46.12	48.62		
	Truck	58.49	52.28	52.13		
	Tricycle	45.89	29.05	26.40		
	Awning-tricycle	32.98	23.07	18.78		
	Bus	64.45	61.24	61.72		
	Motor	55.08	43.79	42.98		
YOLOv4-ours	All	62.71	51.20	52.76	44.79	0.028
	Pedestrian	68.98	56.04	60.59		
	People	58.45	36.89	39.14		
	Bicycle	50.72	29.42	29.81		
	Car	77.16	82.41	83.72		
	Van	65.84	52.63	56.59		
	Truck	68.11	59.57	62.95		
	Tricycle	55.14	39.41	38.19		
	Awning-tricycle	44.53	33.78	30.92		
	Bus	73.73	70.23	72.56		
	Motor	64.48	51.58	53.15		

Similarly, on the S2TLD dataset, our model has better detection performance compared to the YOLOv4 model, as shown in Table 6. In addition, our model has high accuracy in the yellow light category, which is easily confused with the red light. The improved model has better precision and recall for small objects in images, has fewer parameters, and meets the detection speed of real-time detection.

**Table 6:** Compare the detection performance, detection speed, number of parameters of YOLOv4 and the proposed model (YOLOv4-ours) on the S2TLD dataset.

Model	Class	P/%	R/%	mAP/%	Parameter/million	t/s
YOLOv4	All	92.50	94.80	95.32	63.96	0.025
	Red	97.16	96.54	98.15		
	Yellow	86.74	96.08	93.78		
	Green	94.97	96.10	97.33		
	Off	89.20	93.84	92.52		
	Wait_on	94.45	91.43	94.79		
YOLOv4-ours	All	93.35	96.10	96.98	44.77	0.028
	Red	95.88	97.21	98.54		
	Yellow	96.13	97.31	98.66		
	Green	94.54	96.48	97.88		
	Off	84.52	94.32	93.79		
	Wait_on	95.69	95.17	96.03		

### Comparison with other object detection models

In this section, we compare the detection performance of the improved models with the current mainstream real-time object detection models, including Faster-RCNN (Ren et al., 2015), YOLOv4, YOLOv5L, and YOLOR (Wang, Yeh & Liao, 2021), on these two datasets, and the experimental results are shown in Tables 7 and 8. Since we first lightened the YOLOv4 model to obtain a Version-1 model with faster detection, higher mAP, and fewer parameters and improved the feature fusion module on top of this, our final model still has fewer parameters and has the best mAP compared to other algorithms. Our improved YOLOv4 model also inherits the advantages of the fast detection speed of the YOLO series algorithms. The detection speed of a single image is 0.034 s faster than the Faster-RCNN model, which can meet the criteria of real-time object detection and is more suitable for real-time detection of small objects in complex backgrounds compared to other algorithms. In addition, we compare the experimental results with existing evaluation results based on the datasets used in this article, including those on the three models Cascade R-CNN (Cai & Vasconcelos, 2018), RetinaNet (Lin et al., 2017b) and FPN, as shown in Tables 7 and 8. Among them, regarding the VisDrone2019 dataset, we compared the evaluation results of the advanced models provided by The VisDrone team (Du et al., 2019). Regarding the S2TLD dataset, we compared the evaluation results of the advanced models provided by the authors of this dataset (Yang et al., 2022). It can be found that the detection accuracy of our proposed model is better than these existing evaluation results.

**Table 7:** Comparison of the performance of different detection algorithms on the VisDrone2019 dataset.

Model	mAP/%	Parameter/million	t/s
YOLOv4	42.79	63.99	0.025
Faster-RCNN (ResNet50)	33.24	70.55	0.062
YOLOv5l	42.10	46.16	**0.020**
YOLOR	43.17	52.51	0.023
FPN	32.20	—	—
Cascade R-CNN	31.91	—	—
RetinaNet	21.37	—	—
YOLOv4-ours	**52.76**	**44.79**	0.028

**Note:**

The best results are in bold.

**Table 8:** Comparison of the performance of different detection algorithms on the S2TLD dataset.

Model	mAP/%	Parameter/million	t/s
YOLOv4	95.32	63.96	0.025
Faster-RCNN (ResNet50)	91.51	70.52	0.062
YOLOv5l	94.79	46.13	**0.020**
YOLOR	94.64	52.49	0.023
FPN	94.93	—	—
RetinaNet	93.25	—	—
YOLOv4-ours	**96.98**	**44.77**	0.028

**Note:**

The best results are in bold.

## Conclusions

To improve the performance of small object detection in the context of complex road environments, we propose an approach to improve the YOLOv4 network. Firstly, we used the CSP structure and SiLU activation function in the neck of YOLOv4 to ensure the accuracy of the model while reducing the model parameters. Then, we tried to change the feature fusion module structure to obtain richer location information by introducing additional shallow feature information from the backbone network into the feature fusion module and using a new detection head that is more suitable for detecting small objects, thus improving the performance of the model. We have also tried to improve the feature fusion module by using a simplified BiFPN structure, which allows the model to receive more layers of feature information during feature fusion and enhances the fusion weight of useful information, thus improving model performance. Moreover, we have added an attention mechanism to the feature fusion module to improve the model's ability to focus on spatial location relationships and inter-channel relationships in feature information. As a result, our network achieved a high mAP of 52.76% and 96.98% on two small object datasets, the VisDrone2019 and the S2TLD, respectively, an improvement of 9.97% and 1.66% over the original YOLOv4 network, while the number of parameters was only 70% of the original YOLOv4 network. The improved model achieves the highest mAP compared to some of the existing state-of-the-art models, and the detection speed can meet the criteria for real-time detection. In addition, the proposed model has only 44M parameters, which is easier to deploy to mobile devices than other advanced models in practical applications. The current work provides an effective method for small object detection in complex environments, which can be extended to detection tasks such as drone cruises and unmanned vehicles.

Although our model has better performance in detecting small objects with complex backgrounds, it still reduces detection speed while improving detection accuracy, and subsequent research work can further reduce the model parameters and improve the detection speed through model compression methods such as model pruning. In another aspect, the model has limited feature information about small objects that can be extracted from the original input image, and subsequent research work can use generative adversarial networks to generate high-resolution images to further improve small object detection performance.

## Additional Information and Declarations

### Data Availability

The following information was supplied regarding data availability: The data are available at GitHub: https://github.com/VisDrone/VisDrone-Dataset, https://github.com/Thinklab-SJTU/S2TLD. These datasets are available at Zenodo: - Gang Liu. (2022). Repartition of part of visdrone2019 dataset [Data set]. Zenodo. https://doi.org/10.5281/zenodo.7355398. - Gang Liu. (2022). Repartition of S2TLD dataset [Data set]. Zenodo. https://doi.org/10.5281/zenodo.7352960. - Gang Liu. (2022). Correlation weights of small target detection papers. Zenodo. https://doi.org/10.5281/zenodo.7359112. We used part of the Visdrone2019 dataset provided by Zhu (https://github.com/VisDrone/VisDrone-Dataset) and all of the S2TLD dataset provided by Yang (https://github.com/Thinklab-SJTU/S2TLD). We redivided the two datasets into the training set, the validation set, and the test set in a ratio of nearly 7:2:1. The code are available at Zenodo: Gang Liu. (2022). the code of my manuscript. Zenodo. https://doi.org/10.5281/zenodo.7370140.
